# Tunable Terahertz Deep Subwavelength Imaging Based on a Graphene Monolayer

**DOI:** 10.1038/srep46283

**Published:** 2017-04-11

**Authors:** Heng-He Tang, Tie-Jun Huang, Jiang-Yu Liu, Yunhua Tan, Pu-Kun Liu

**Affiliations:** 1 School of Electronics Engineering and Computer Science, Peking University, Beijing, 100871, China

## Abstract

The resolution of conventional terahertz (THz) imaging techniques is limited to about half wavelength, which is not fine enough for applications of biomedical sensing and nondestructive testing. To improve the resolution, a new superlens, constructed by a monolayer graphene sheet combining with a grating voltage gate, are proposed in this paper to achieve deep super-resolution imaging in the THz frequency range. The main idea is based on the Fabry-Perot resonance of graphene edge plasmon waves. By shaping the voltage gate into a radial pattern, magnified images of subwavelength targets can be obtained. With this approach, the finest resolution can achieve up to λ/150. Besides, the superlens can be conveniently tuned to work in a large frequency band ranging from 4.3 THz to 9 THz. The proposal could find potential applications in THz near-field imaging systems.

In recent years, terahertz (THz) imaging applications^1^ have received constantly growing interest due to the capability of THz waves to penetrate a wide range of materials^2^. However, the resolution of conventional THz imaging techniques are limited to millimeter range^3^, owing to the evanescent decay of high spatial frequencies away from their source, the fact of which is also well known as the diffraction limit^4^. Even though THz near-field scanning imaging systems^5^,^6^,^7^ can achieve nanometer scale spatial resolution by using probes with sharp tips, the whole process of image reconstruction is troublesome and time-wasting since the point by point detection needs to be performed. The proposal of optical superlens^8^,^9^,^10^ constructed by negative index metamaterials or plasmonic materials (such as silver) targets to realize real-time super-resolution imaging. The collective oscillating of electrons in silver, known as surface plasmon polaritons (SPPs)^11^, would result amplification of the evanescent spatial frequencies. Nevertheless, experiments show that SPPs in silver become very weak^12^ in the THz frequency range. This is the main obstacle when we extend the working frequency of superlens and hyperlens^13^,^14^ to THz frequencies. Since the internal images of objects can be glimpsed owing to the penetrability of THz waves, realizing super-resolution imaging in the THz frequency range shows its special advantages on applications such as biomedical sensing^15^ and nondestructive testing^16^. By periodically perforating subwavelength square holes^17^ or embedding subwavelength grooves^18^ in metals, mimicking SPPs can be generated at THz frequencies. Structures based on this kind of metastructures have been well designed to break the diffraction limit^19^,^20^.

Graphene, emerged as the most promising 2D plasmonic material in the THz to mid-infrared frequency range^21^, has become an ideal candidate for designing plasmonic devices^22^. Compared with the mimicking SPPs, graphene plasmons show the superiority on tunability by chemical doping or gate voltage^23^. By dynamically tuning the conductivity of graphene, a planar gradient index graphene-based lens is designed to control the SPPs beam deflections^24^ or focusing^25^. In addition, significant enhancements of evanescent waves for near-field subwavelength imaging are demonstrated by a monolayer graphene^26^ and layered graphene^24^,^27^,^28^. More importantly, hyperlenses constructed by a modulated graphene monolayer^29^ or graphene/dielectric multilayers^30^ are also explored to realize subwavelength imaging at THz frequencies. But the hyperlenses cannotrealize frequency tuning conveniently, this is the inherent drawback of the constructed structures with hyperbolic isofrequency contour.

In this paper, a new graphene-based superlens is proposed to break the diffraction limit in the THz frequency range. The superlens is composed by a monolayer graphene sheet and a metallic grating voltage gate. The working mechanism of the superlens relies on Fabry-Perot resonance of graphene edge plasmon waves. The working frequency can be conveniently tuned over a large range by changing the gate voltage. Furthermore, magnified images of subwavelength targets can be obtained if we shape the voltage gate into a radial pattern. A deep resolution of 400 *nm* is demonstrated, which indicates our proposal could be suitable for biomedical applications.

## Results

### Graphene Plasmons

**Figure 1:**
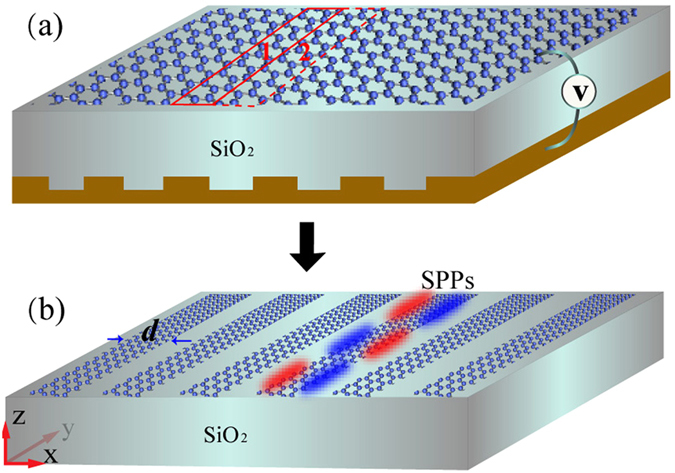
Schematic of the near-field superlens. (**a**) is the real model. A monolayer graphene sheet is supported on the SiO_2_ substrate and the conductivity of the sheet is periodically tuned by a grating voltage gate. (**b**) is the equivalent model at a given frequency. The graphene ribbons correspond to the regions in the graphene sheet with high conductivity.

The schematic of the near-field superlens is illustrated in Fig. 1(a). A monolayer graphene sheet is supported on a SiO_2_ substrate. The permittivity of the SiO_2_ is 3.9. On the back of the substrate, there is a metallic grating voltage gate. Different separations between the graphene and the metallic surface of the grating lead to non-uniform modification of the carrier density, which eventually generates the periodical conductivity along the x direction. This is now a common method used for tuning the conductivity of graphene^31^. As we know, the complex conductivity of graphene, expressed as *σ*_*g*_ = *σ*_*g,r*_ + *iσ*_*g,i*_, depends on angular frequency *ω*, electron relaxation time *τ* representing the loss mechanism, temperature *T*, and chemical potential *μ*_*c*_. The imaginary part of the conductivity determines the propagation of SPPs waves, the real part of the conductivity represents the losses. When *σ*_*g,i*_ > 0, a single free-standing layer of graphene effectively behaves as a very thin “noble metal” layer with negative permittivity. Hence, the graphene sheet can surppot a transverse-magnetic (TM) polairized SPPs surface wave. The dispersion of the two dimensional graphene surface plasmon (2DGSP) supported on the sheet can be expressed as^32^


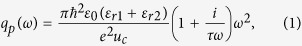


where *ε*_*r*1_ and *ε*_*r*2_ are the relative permittivity of the dielectrics above (here it is the air) and under the graphene sheet, *ε*_0_ is the permittivity in vacuum, *e* is the electron charge, *ħ* is the Plank’s constant. *q_p_* represents the in-plane wave-vector of the plasmon waves.

For the model in Fig. 1(a), the spatial period of the conductivity is *p*, corresponding to the period of the grating. We assume the conductivities of the two adjacent regions (regions 1 and 2) are respectively *σ*_*g*1_ and *σ*_*g*2_. If the conditions that Im(*σ*_*g*1_) > 0 and Im(*σ*_*g*2_) < 0 or Im(*σ*_*g*2_) ≪ Im(*σ*_*g*1_) are fulfilled, Fresnel reflection will occur on the boundary of the two regions. Hence, the surface waves will be confined to a fictitious boundary^33^,^34^. It gives a simple explanation of how the SPPs edge mode being excited. Different from the 2DGSP, the edge mode has maxima of intensity near the boundaries. Such one-dimensional (1D) excitations are generic for thin lossy metal films with finite width, although their dispersion depends on the thickness and the dielectric environment of the edge^35^. The 1D edge mode is also theoretically and experimentally observed on a graphene ribbon or a ribbon array^36^,^37^. Actually, a graphene ribbon can be either a real graphene strip or something that is “virtually” created by spatially varying external gates acting on a graphene sheet, as proposed in ref. 34. In this way, the physical model of the superlens is equivalent to the model in Fig. 1(b) at a given frequency point. The graphene ribbon array in Fig. 1(b) corresponds to the regions in the graphene sheet with the conductivity being *σ*_*g*1_. The width of the ribbons is *d*, and the duty ratio is 0.5. Below, we will use this equivalent model to present the analysis. Although the microscopic structure of graphene edges modifies the electronic spectrum significantly, the excitation of the edge mode depends only on total charges and total currents. When the separation between the Femi energy and the Dirac point is much larger than the temperature or relaxation energy, the nature of what kind of edge does not affect the calculations reported here^34^.

It has been well demonstrated that the wavelengths of the 1D edge plasmons (*λ*_*ep*_) and the 2D surface plasmons (*λ*_*p*_) differ by a universal numerical factor^37^, i.e. *λ*_*ep*_ = *C*_0_·*λ*_*p*_, where *C*_0_ is the factor, *λ*_*p*_ = 2*π*/Re(*q*_*p*_). The value of *C*_0_ increases with the width of the ribbon, but we always have *C*_0_ < 1. The physical reason for the smaller wavelength of the edge plasmons compared to the 2D ones is the effective reduction of the Drude weight at the edges, where free carriers exist only on “one side”. The electric field of the edge plasmons decays exponentially away from the edge. For a given ribbon width, high order modes, which are actually the hybrids of 1D edge mode and 2DGSP mode, can be excited at large frequencies. But the 2DGSP mode disappears at low frequencies. Each of the high order modes has a cutoff frequency. Hence, there is always a frequency band for the single fundamental mode (i.e. the 1D edge mode) operation. The narrower the width of the ribbon is, the broader the frequency band will be. In the frequency range we concerned, the condition of single fundamental mode operation is always satisfied by cutting down the width of ribbons to nanometer scale (much smaller than the wavelength).

The dispersion of graphene ribbons has been extensively studied by a variety of theories such as the theory of the hydrodynamics of electron-hole liquid^33^. A simple electrostatic scaling law reported in ref. 38 can also be used to quantitatively obtain the universal plasmon dispersion curves. The plasmon frequencies have an analytical expression of


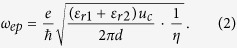


In equation (2), *η* is the scaling parameter which is only a function of the product *q*_*ep*_ *d*, where *q*_*ep*_ is the wave-vector of the edge mode. For an arbitrary *d*, the value of *η* could be analytically fitted by the simulated data of a given ribbon. By this way, we can obtain the dispersion curves corresponding to different graphene ribbons.

**Figure 2:**
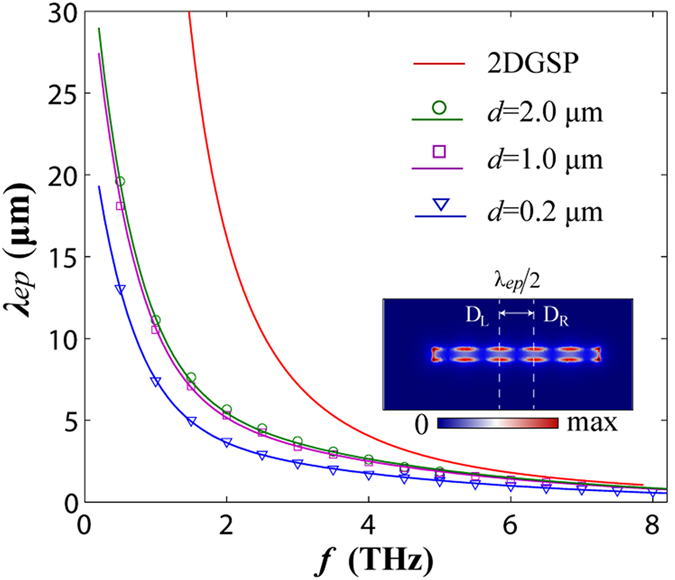
Dispersion of a graphene sheet and graphene ribbons with different widths. The solid lines are the theoretical results. The circular, the square and the triangular dots are the simulated results corresponding to *d* = 2.0 μm, *d* = 1.0 μm and *d* = 0.2 μm, respectively. The inset shows the profile of the edge mode.

The numerical calculations are performed with the help of the finite elements methods using Comsol Multiphysics. The parameters of the graphene ribbon are chosen to be *u*_*c*_ = 0.15 eV, *τ* = 0.5 ps at room temperature T = 300 K (the values of *τ* and T keep no change throughout the paper). These parameters are typical for graphene plasmons observed in THz to infrared frequency range^32^,^34^. The simulated plasmon wavelength *λ*_*ep*_ is calculated by 2(D_R_-D_L_), D_R_ and D_L_ are respectively the right and the left positions of two adjacent peaks, as illustrated in the inset of Fig. 2. Both the simulated (dots) and fitting (solid lines) dispersion curves for three ribbons with *d* = 0.2 *μm*, 1 *μm* and 2 *μm* are presented in Fig. 2. We notice that, at a given frequency, if *d* decreases, the wavelength of the edge mode becomes shorter, which means that the 1D localized mode is more confined to the rims (the decrease of *λ*_*ep*_ corresponds to the increase of *q*_*ep*_, therefore, the surface wave decays faster). As we can observe, in the lower frequency range, the gap of the plasmon wavelengths between the edge mode supported on the nanoribbon and the 2DGSP (shown with red solid line in Fig. 2) supported on a graphene sheet is apparent. As shown in Fig. 2, the analytical fitting curves match well with the simulated data.

### Near-Field Super-Resolution Imaging

For the graphene nanoribbon array shown in Fig. 1(b), each of the nanoribbons could be regarded to a transmission path of the edge mode. The whole system is similar to metal films with a periodic arrangement of cut-through slits^39^. The nanoribbons correspond to the subwavelength slits. The presences in the silts are TEM modes, but the presences on the nanoribbons are the edge modes. The electric field of the edge mode is expressed as 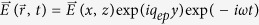, where 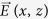
represents the transverse mode pattern. When a monochromatic plane wave with its *x*-direction wave-vector being *k*_*x*_ impinges upon the front interface of the nanoribbon array, the edge modes will be excited with different initial phases (could be known by (*k*_*x*_ ± 2*nπ/p*)·*m*′*p*) and propagate along each of the nanoribbons, *n* and *m′* are both integers and refers to the *n*-order harmonic and the *m*′*-*th nanoribbon respectively counting from left to right. One of the electric field components on the *m-*th nanoribbon can be mathematically expressed as


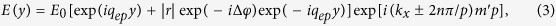


in which, *r* and Δ*φ* are respectively the reflection coefficient and the phase jump of the reflected surface wave at the boundary, Δ*φ* = tan^−1^[Im(*r*)/Re(*r*)], *E*_0_ is related to the coupling strength. As can be observed, regardless of the angle of the oblique incidence (including the evanescent harmonics), the incident plane waves will always be coupled to the graphene edge mode with the same longitudinal wave-vector, i.e. *q*_*ep*_. Hence, the phase accumulations of any incident waves are the same at the emergence interface of the graphene nanoribbon array. The scattering waves of subwavelength targets are an integration of all spatial harmonics with corresponding amplitudes and phases. In order to obtain an undistorted image of the targets, the amplitude and the phase at the emergence interface must keep synchronous with the amplitude and phase at the incoming interface. Under this requirement, we can deduce the following equation after considering the boundary condition at both the incoming and the emergence interfaces


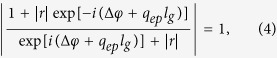


where *l*_*g*_ is the length of the nanoribbons. Equation (4) can be satisfied if


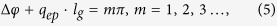


Equation (5) is the Fabry-Perot resonance condition^30^,^40^. We conclude that the subwavelength targets can be resolved at the emergence interface only when the Fabry-Perot resonance of the edge mode is set up. After substituting Δ*φ* and *q*_*ep*_ into equation (5), we can calculate the value of *l*_*g*_. An analytical method to determine Δ*φ* has been reported in ref. 40, and it is claimed that Δ*φ* is approximately constant during a broadband frequency range. For simplicity here, we can determine Δ*φ* by numerical simulation. When the parameters of the nanoribbons are *u*_*c*_ = 0.15 eV and *d* = 0.2 *μm*, we get Δ*φ *≈ 0.54*π* in the 4 THz-9 THz frequency range. When the working frequency is chosen to be *f* = 5 THz, we have *q*_*ep*_ = 46.12*k*_0_, in which *k*_0_ is the wave-vector in free space. The conductivity of the graphene nanoribbons at 5 THz is *σ*_*g*1_ = 0.035 + *i*0.56 mS. For *m* = 2, we obtain *l*_*g*_ = 0.95 *μm*.

**Figure 3:**
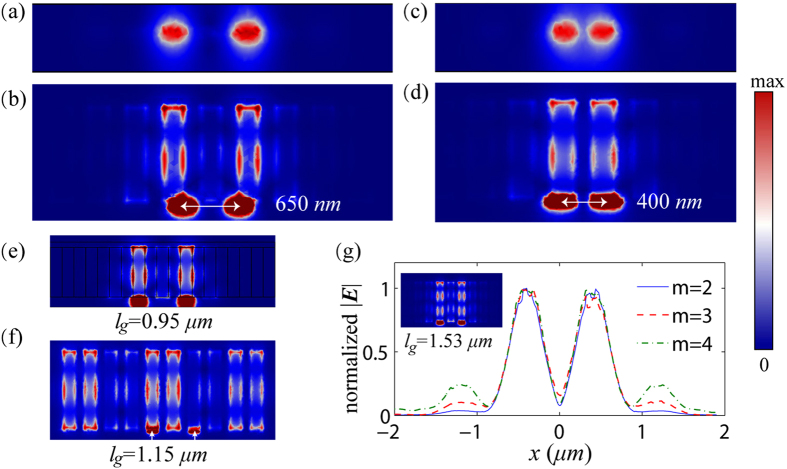
Near-field super-resolution imaging. (**a**,**b**) are respectively the electric field mapping on x-z plane and x-y plane. The two sources are separated by 650 nm. (**c**,**d**) are respectively the electric field mapping on x-z plane and x-y plane. But the distance of the two sources changes to be 400 nm. (**e**) presents the simulation on a monolayer graphene sheet. The two sources are separated by 800 nm. (**f**) presents the simulated electric field when l_g_ changes to be 0.95 μm. (**g**) is the normalized image of the line current sources when the length of the graphene ribbons are *l*_*g*_ = 0.95 μm, 1.53 μm, 2.12 μm, respectively corresponding to the 2^nd^, the 3^rd^ and the 4^th^ Fabry-Perot resonance. The electric field mapping of the 3^rd^ Fabry-Perot resonance is shown in the inset.

Next, numerical simulation using Comsol is performed to demonstrate the super-resolution imaging of the superlens. In Fig. 3(a), the near-field 2D images (electric field mapping on the *x-z* plane) of two targets are presented. Here, the targets are surface current sources with their directions parallel to *z*-axis. The *x*-direction width of the targets is 250 *nm*. The two targets are separated by 650 *nm* and located 60 *nm* away from the incoming interface. The 2D images are detected at the distance of 60* nm* away from the emergence interface. As the above theory predicted, the two subwavelength targets are able to be perfectly distinguished in the near-field by the graphene superlens. The *x-y* plane electric field mapping above the graphene is shown in Fig. 3(b), from which we can observe the transmissions of the edge modes. In this case, the *λ*/92 imaging resolution is demonstrated. The near-field 2D images of the two incident targets which are separated 400 *nm* are illustrated in Fig. 3(c), the *x-y* plane electric field mapping is illustrated in Fig. 3(d). The two obvious peaks corresponding to the two targets also demonstrate the performance of the superlens. The imaging resolution in this case is *λ*/150. Theoretically speaking, this is the ultimate resolution of the designed graphene superlens because the resolution cannot be smaller than the period of the nanoribbon array. To improve the resolution, one can further narrow the width of the nanoribbons.

Even though all of the above simulations are done on the graphene nanoribbons array, the same results will be obtained if we replace the graphene nanoribbons with a monolayer graphene sheet tuned by a grating voltage gate. At 5 THz, we have Im(*σ*_*g*1_) = 0.56 mS and Im(*σ*_*g*2_) = 0.0012 mS. The two targets are separated by 800 *nm*. As shown in Fig. 3(e), the *x-y* plane electric field mapping in this case is almost the same as Fig. 3(b). To further verify the role of the Fabry-Perot resonance, we simulate a case of that the Fabry-Perot resonance condition is not satisfied. As presented in Fig. 3(f), the *y*-direction length of the graphene sheet (or nanoribbons) is *l*_*g*_ = 1.15 *μm*, the images of the two targets are disturbed. Hence, we cannot simply regard each of the nanoribbons as a field probe. When *l*_*g*_ = 1.53 *μm*, as shown in the inset of Fig. 3(g), the images are reconstructed again because the 3^rd^ order (*m* = 3) Fabry-Perot resonance is established. The normalized 1D images corresponding to the 2^nd^, the 3^rd^ and the 4^th^ order Fabry-Perot resonance, plotted along a *x*-direction line which is 60 *nm* away from the emergence interface, are also shown in Fig. 3(g). The lengths of the superlenses are *l*_*g*_ = 0.95 *μm*, 1.53 *μm*, 2.12 *μm*, respectively corresponding to *m* = 2, 3, 4 in equation (5). Now, we can see the key role of the Fabry-Perot resonance in determining the image reconstruction.

### Frequency Tuning

Apart from the deep subwavelength resolution, another advantage of our designed graphene superlens is that it can be easily tuned to work over a broadband frequency range. As we have pointed out, what dominates the edge modes propagation on the graphene sheet is *σ*_*g*1_, hence, it must be accurately controlled. But there is a wide range of values for *σ*_*g*2_ to insure the Fresnel reflection of surface wave. A typical value range of Im(*σ*_*g*2_) can be −0.5 mS ~ 0.01 mS. Therefore, the performance of the superlens is not sensitive to *σ*_*g*2_. We only need to modulate the value of *σ*_*g*1_ for frequency tuning. This is totally different from those hyperbolic-dispersion-based super-resolution imaging mechanisms which need to accurately control *σ*_*g*1_ and *σ*_*g*2_ simultaneously^29^,^41^. Actually, if we do not take into account the difficulty of preparing the graphene nanoribbon samples and adding the voltage gates on each of the ribbons, we can also use the model in Fig. 1(b) to realize frequency tuning.

**Figure 4:**
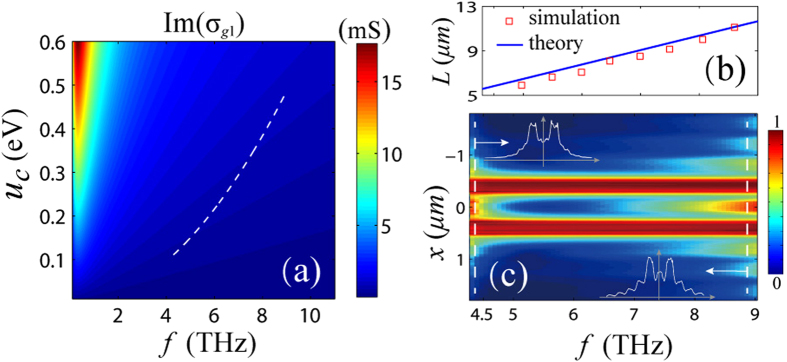
Numerical demonstrations of frequency tuning. (**a**) is the color map of the imaginary part of the conductivity as a function of *f* and *u*_*c*_. The width of the ribbons is *d* = 0.2 μm. The white dashed line in the figure illustrates the frequency tuning path, ranged from 4.3 THz~9 THz. (**b**) presents the theoretical (solid line) and simulated (square dots) losses as a function of the tuning frequency. The losses are characterized by the propagation length of the edge mode. (**c**) is the normalized image as a function of the tuning frequency. The image curves at the low (4.4 THz) and high (8.8 THz) spectral window are presented in the inset.

As mentioned above, the principle of realizing super-resolution imaging is to set up the Fabry-Perot resonance. To ensure this, we only need to adjust the gate voltage to get a identical *q*_*ep*_ at different working frequency when *l*_*g*_ is fixed. To achieve this, *u*_*c*_ must follow a linear relation with the square of *f*, i.e. *u*_*c*_ = *α*·*f*^2^, in which *α* is a positive constant, known from equation (2). When the working frequency is tuned from 4.3 THz to 9 THz, the calculated *u*_*c*_ must increase from 0.111 eV to 0.486 eV accordingly, as shown in Fig. 4(a). The corresponding conductivity of the graphene is from 0.0356 + *i*0.481 mS to 0.0357 + *i*1.01 mS. The upper limit of the frequency range is a conservative estimation. If high quality graphene with large carrier density (could be achieved by highly doping) is available, a higher working frequency can be expected.

The lower limit of the frequency range is mainly subjected to the losses. The losses can be characterized by the propagation length of the edge mode, which should refer to the imaginary part of the wave-vector. In the working frequency range, the propagation length can be roughly calculated by *L* = [Im(*q*_*p*_)]^−1^. As shown in Fig. 4(b), the propagation length increases from 5.48 *μm* to 11.66 *μm* if we tune the frequency from 4.3 THz up to 9 THz. The square dots are the simulated results of the 200 *nm* nanoribbon. The theoretical and simulated results are in good agreement. The losses decrease when the frequency increases. The minimum propagation length is much larger than the length of the nanoribbons. In Fig. 4(c), we plot the color mapping of the normalized images of two sources as a function of frequency. The two sources are separated by 800 *nm*. The images are detected along a *x*-direction line which is 60 *nm* away from the emergence interface. From 4.3 THz to 9 THz, the two peaks corresponding to the two targets can always be distinguished. The image curves at the low (4.4 THz) and the high (8.8 THz) spectral window are also presented in the inset of Fig. 4(c). The two peaks in the two curves are still obvious although the qualities of the images are slightly deteriorative.

### Magnifying subwavelength targets

**Figure 5:**
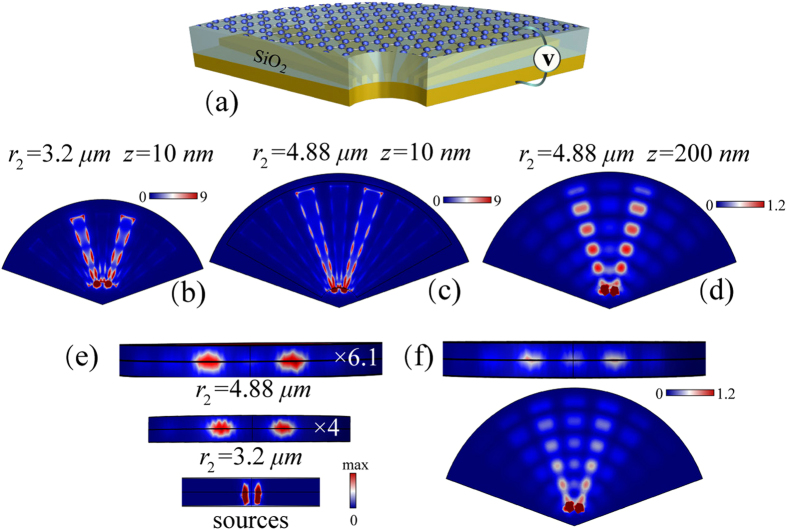
Magnified super-resolution imaging. (**a**) is the schematic of the superlens. The voltage gate is a fan-shaped metallic grating. (**b**) is the simulated electric fields mapping at the z = 60 nm plane for the cases of r_2_ = 3.2 μm and 4.88 μm. (**d**) is the electric field mapping at z = 200 nm plane, corresponding to the case r_2_ = 4.88 μm. The sources and the 2D images at the output curved plane are shown in (**e**). (**f**) presents the simulation corresponding to (**d**) but with loss being considered. The two line sources are separated by 0.8 μm.

In practical applications, we always hope magnified images could be obtained so that two objects could be distinguished by conventional imaging system. Hyperlenses which allow magnification of deep subwavelength scale objects have been proposed by using anisotropic materials with hyperbolic dispersions^14^. Our further study finds that, the goal can be realized by shaping the grating metallic gate into radial pattern, as shown in Fig. 5(a). By doing this, graphene ribbons with gradual increased width can be built, where *r*_1_ and *r*_2_ are respectively the inner and the outer radius of the ribbons, *θ* is the angle of the ribbon. The basic principle is the same as above. But in this case, the plasmon wavelength of edge mode is no longer constant at a given frequency because the width of each ribbon is gradually broadening. Hence, the Fabry-Perot resonance condition now becomes


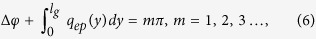


in which, *l*_*g*_ = *r*_2_ - *r*_1_. When the working frequency is 8 THz, the conductivities of the graphene ribbons become *σ*_*g*1_ = 0.0357 + *i*0.898 mS and *σ*_*g*2_ = 2.38×10^−4^ + *i*5.98×10^−3^ mS. The magnification factor of image can be calculated by γ = *r*_2_/*r*_1_. If *r*_1_ is fixed, increasing the value of *r*_2_ would enlarge the magnification factor. Nevertheless, the value of *r*_2_ can only be discrete because the Fabry-Perot resonance condition must be fulfilled. To prove it, we choose *r*_1_ = 0.8 *μm, θ* = 8°. The duty ratio of the gate is 0.5. Two surface sources separated by 800 *nm* are located 60 *nm* away from the incoming interface. The simulated electric field mapping at the *z* = 10 *nm* plane for the cases of *r*_2_ = 3.2 *μm* and 4.88 *μm* are respectively shown in Fig. 5(b,c). As we can note, the two targets are resolved and gradually magnified with the help of the edge modes. In Fig. 5(d), the electric field mapping at *z* = 200 *nm* plane is also presented. Because of the evanescent decay of the fields in the *z* direction, the strongly confined edge modes become 2D “bulk” modes. As shown in Fig. 5(e), the space of the two sources is respectively magnified by 4 folds and 6.1 folds in the two cases.

The lossless simulations have perfectly demonstrated the mechanism of realizing magnified super-resolution imaging. But in reality, the compromise should be made between the loss and the magnification factor. As we can see in Fig. 5(f), when the loss is considered in Fig. 5(d), the performance of the superlens is influenced but still competent for targets recognition.

Realizing far-field super-resolution imaging requires a relative long graphene ribbon (≈40 *μm*). Although in our demonstrations, the length (corresponding to the magnification factor) is not sufficient for compressing the evanescent spatial spectrum of the deep subwavelength targets into propagating spectrum, far-field super resolution imaging could also be predicted if we lower the designed maximum resolution (correspondingly, reduce the required length of ribbons) or mitigate the loss influence. The method of realizing frequency tuning for this superlens is the same as above, therefore, we will not cover it again.

**Figure 6:**
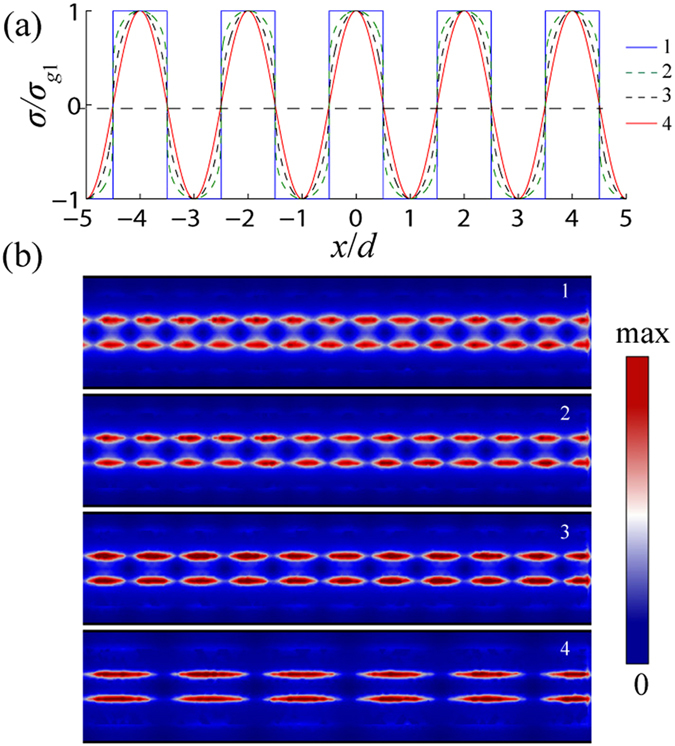
Modeling the influence of non-ideal boundaries between the high and low carrier density regions. (**a**) shows the four conductivity profiles considered in modeling. (**b**) shows the corresponding edge plasmon modes in the four cases.

In reality, the boundaries between the high and low carrier density regions cannot be made infinitely sharp electrostatically. To discuss the influence, four conductivity profiles are considered, as shown in Fig. 6(a). The first one is the perfect rectangular profile, i.e. the profile we used above. The fourth one fulfills the standard cosine function, which can be expressed by 
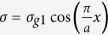
, we imagine this is the worst case. The conductivity profile of the second and third ones can be expressed by


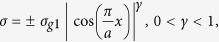


with the *γ* respectively being *γ*_2_ = 0.2 and *γ*_3_ = 0.5. We find that, decreasing *γ* means the profile is closer to the perfect one. The simulated results of the four cases are shown in Fig. 6(b). Even though the conductivity profile is not infinitely sharp, the edge plasmons are also excited. However, the wavelength of the edge mode slowly increases with the conductivity profile getting close to be worse. This is reasonable because the conductivities near the boundaries change with the profile. The increasing of the edge mode wavelength caused by the non-ideal boundaries would introduce error when calculating the Fabry-Perot resonance condition. But the mechanism of near-field super-resolution imaging still holds. Hence, the practical length of the graphene ribbons must be adjusted according to the fabricating error.

The structure of our proposed graphene superlens is simple, losses in the superlens mainly comes from the graphene material. Generally speaking, the losses of graphene is related to the temperature, the electron relaxation time, the chemical potential and the working frequency. The imaging mechanism can be extended to a broadband frequency range (up to infrared). Even though the demonstrated propagation lengths of the edge plasmon modes are limited, the lengths could be further increased by optimzing the parameters of the graphene and the frequency to reduce loss. We believe it is possible for using the graphene superlens in practical applications.

To conclude, we have introduced a new way to realize deep super-resolution imaging in the THz frequency range by using a monolayer graphene sheet tuned by a grating voltage gate. Magnified images of subwavelength targets also can be obtained by shaping the voltage gate. The finest resolution of the superlenses can reach 400* nm*. Furthermore, the superlenses can be tuned to work over 4.3 THz ~ 9 THz frequency range. Owing to the strong confinement of the graphene plasmon edge mode, the images are well resolved in both the *x* and the *z* directions. It indicates that our proposal may be suitable for 2D super-resolution imaging. The mechanism could be extended to the infrared frequency range. The proposal could find potential applications in THz near-field imaging systems.

## Methods

Numerical simulations are performed by the commercial software, Comsol Multiphysics, which is based on the Finite Element Method (FEM). The direct and iterative solvers are employed. The monolayer graphene sheet or ribbon is characterized by surface currents *j*_∥_ = *σ*_*g*_*E*_∥_, where the symbol “∥” means the component is parallel to the surface of the graphene. According to the Kubo formula, the two specific intraband and interband electron transitions contribute to the surface conductivity of a monolayer graphene, which can be calculated from^42^


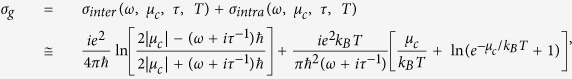


in which, *e* is the electron charge, *ħ* and *k*_*B*_ are the Plank’s constant and the Boltzman’s constant, respectively. The chemical potential depends on the carrier density ns which is controlled by the gate voltage. Notice that for relatively small frequencies(*ħω/μ*_*c*_ ≪ 1), the intraband electron transition dominates the conductivity, hence, In the condition of *μ*_*c*_ ≫ *k*_*B*_T, a simple semiclassical model for conductivity, which possesses a Drude-like expression, is obtained


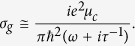


The imaging targets in the simulating models are two rectangular surfaces. The width and the length of the two surfaces are 250 nm and 500 nm, respectively. The excitation sources are surface currents added on both of the two rectangular surfaces. The direction of the currents is perpendicular to the graphene. The minimum size of the grid is smaller than 1/6 of the plasmon wavelength.

## Additional Information

**How to cite this article:** Tang, H.-H. *et al*. Tunable Terahertz Deep Subwavelength Imaging Based on a Graphene Monolayer. *Sci. Rep.* **7**, 46283; doi: 10.1038/srep46283 (2017).

**Publisher's note:** Springer Nature remains neutral with regard to jurisdictional claims in published maps and institutional affiliations.
